# Menopausal Symptom Burden and Self-Reported Poor Sleep Quality Among Japanese Working Women Aged 40 Years and Older: An Exploratory Cross-Sectional Study

**DOI:** 10.3390/medicina62101856

**Published:** 2026-09-25

**Authors:** Makiko Arima, Yoshikuni Edagawa

**Affiliations:** 1 School of Medicine, Showa Medical University, Tokyo 142-8777, Japan; 2 Graduate School of Technology Management, Ritsumeikan University, Osaka 567-8570, Japan; edagaway@fc.ritsumei.ac.jp

**Keywords:** menopause, menopausal symptoms, sleep quality, objective sleep duration, working women, Simplified Menopausal Index, wearable EEG

## Abstract

*Background and Objectives*: Sleep-related complaints are common among women in midlife and may reflect not only insufficient sleep duration but also broader menopausal symptom burden. Although menopausal symptoms and sleep problems are known to be related, less is known about how menopausal symptom domains are associated with self-reported sleep quality among working women, particularly when objective sleep duration is considered. This exploratory cross-sectional study aimed to examine the association between menopausal symptom burden and self-reported poor sleep quality among Japanese working women aged 40 years and older, while accounting for objectively measured sleep duration. *Materials and Methods*: Participants completed questionnaires on sleep quality and menopausal symptoms and underwent objective sleep monitoring using a wearable electroencephalography device. Self-reported sleep quality was assessed using an item corresponding to the Pittsburgh Sleep Quality Index and dichotomized into poor and good sleep quality. Menopausal symptoms were evaluated using the Simplified Menopausal Index, including vasomotor, psychological, and somatic domains, with higher scores indicating greater symptom burden. *Results*: Among 79 participants, 43 reported poor sleep quality and 36 reported good sleep quality. Women with poor sleep quality had higher total and domain-specific SMI scores and shorter objective sleep duration. In logistic regression analyses adjusted for age and objective sleep duration, total SMI score and each symptom domain were associated with poor sleep quality. These associations remained significant in sensitivity analyses excluding the sleep-related SMI item from the total score and psychological domain score. *Conclusions*: Self-reported poor sleep quality among Japanese working women aged 40 years and older may reflect menopausal symptom burden beyond objectively measured sleep duration. The consistency of the findings across sensitivity analyses supports the robustness of this association. Sleep complaints may therefore serve as an accessible signal for recognizing menopausal symptom burden in clinical and occupational health settings.

## 1. Introduction

Sleep-related complaints are common among women in midlife and are increasingly recognized as an important women’s health issue [1,2]. Poor sleep can affect physical and psychological well-being, daily functioning, fatigue, concentration, and quality of life [2,3]. Among working women, sleep-related problems may also impair daytime functioning and work performance, thereby affecting overall well-being and the ability to maintain a healthy work–life balance [4].

This issue is particularly relevant in Japan, where labor force participation among midlife women is high. In 2024, the labor force participation rate among Japanese women aged 35–44 and 45–54 years was approximately 89–91%, and participation among women aged 55–64 years had increased to 72.1% [5]. Thus, sleep and menopausal symptoms in midlife women are increasingly relevant not only to individual health but also to sustained participation in the workforce. The menopausal transition is accompanied by a range of physical and psychological symptoms. Vasomotor symptoms, such as hot flashes and sweating; psychological symptoms, including anxiety and mood changes; and somatic symptoms, such as fatigue and bodily discomfort, may all contribute to sleep-related complaints [2,6]. Previous studies have reported associations between menopausal symptoms and sleep problems during the menopausal transition [7]. However, menopausal stage alone may not fully explain sleep-related complaints, because symptom burden varies widely among women. Therefore, focusing on the severity and domains of menopausal symptoms may provide clinically useful information beyond menopausal status itself.

Subjective sleep quality is an important patient-reported indicator, but it does not necessarily correspond directly to objectively measured sleep duration. Women may report poor sleep quality even when sleep duration alone does not fully explain their sleep experience. Previous studies have noted discrepancies between subjective and objective sleep measures, suggesting that perceived sleep quality may reflect broader physical and psychological conditions in addition to sleep duration [8,9,10]. In this context, self-reported poor sleep quality may be an accessible signal of broader menopausal symptom burden among working women in midlife.

Although the association between menopausal symptoms and sleep problems has been reported, less is known about how menopausal symptom burden and specific symptom domains are associated with self-reported sleep quality among working women. In particular, it remains unclear whether these associations are explained by objectively measured sleep duration. Clarifying this relationship may help clinicians and occupational health professionals recognize sleep complaints not only as sleep-related problems but also as potential indicators of broader menopausal symptom burden.

Sleep quality in midlife women may also be influenced by factors such as age, body mass index, menopausal status, work-related factors, psychological symptoms, and lifestyle characteristics, highlighting the need to consider the broader context when examining the relationship between menopausal symptoms and sleep.

The aim of this exploratory cross-sectional study was to examine the association between menopausal symptom burden and self-reported poor sleep quality among Japanese working women aged 40 years and older, while accounting for objectively measured sleep duration. We also explored associations between specific menopausal symptom domains and self-reported poor sleep quality.

## 2. Materials and Methods

### 2.1. Study Design and Participants

This exploratory cross-sectional analysis used baseline questionnaire data together with average objectively measured sleep duration obtained during the home monitoring period that included approximately two weeks of scheduled monitoring following an initial familiarization period. Although the original study included repeated questionnaire assessments at baseline, Week 1, and Week 2, the primary analyses in the present study focused on baseline menopausal symptom and subjective sleep-quality measures in relation to average objective sleep duration during the monitoring period. Therefore, the present study was treated as an exploratory cross-sectional analysis. The original study was conducted between September 2024 and October 2025 among Japanese working women aged 20–60 years. Participants were recruited through multiple community and professional channels, including women’s health organizations, professional networks, and public invitations. Data collection was conducted in five separate cohorts, with approximately 20 participants in each cohort.

The original study targeted 100 participants, and 92 participants completed the relevant questionnaire items and objective sleep monitoring. For the present analysis, we restricted the analytic sample to participants aged 40 years and older in order to focus on women in midlife, for whom menopausal symptom burden is more clinically relevant. Participants younger than 40 years were excluded from the present analysis. The final analytic sample included 79 Japanese working women aged 40 years and older.

Participants with incomplete questionnaire data were excluded from the relevant questionnaire-based analyses. Objective sleep duration was available for 68 of the 79 participants included in the present analysis. For the remaining 11 participants, total sleep duration could not be obtained because of a device-type-specific output limitation, as described below.

The questionnaire assessed demographic characteristics, employment status, menstrual status, menopausal symptoms, subjective sleep quality, sleep-related variables, and the Pittsburgh Sleep Quality Index (PSQI).

This study was approved by the Institutional Review Board of Showa Medical University on 1 July 2024 (approval number: 2024-107-A). Electronic informed consent was obtained from all participants prior to participation. This study was conducted as part of the project “Evidence Development of the Simplified Menopausal Index (SMI) and Its Association with Work Productivity”, funded by the Japan Agency for Medical Research and Development (AMED) under the program “Establishing R&D Infrastructure toward Social Implementation of Prevention and Health Promotion”. Objective sleep monitoring was incorporated into the project to complement questionnaire-based assessments with objectively measured sleep parameters.

### 2.2. Measurement and Survey Measures

#### 2.2.1. Objective Sleep Monitoring

Objective sleep duration was measured using a simplified electroencephalography (EEG) device, the Philips^®^ SmartSleep^®^ (Philips Japan, Ltd., Tokyo, Japan), during a home monitoring period that included an initial familiarization period of approximately one week followed by approximately two weeks of scheduled monitoring. Participants were instructed to wear the headband-type wearable device each night from bedtime until awakening throughout the monitoring period. The device non-invasively measured brain activity during sleep using embedded EEG sensors. Recorded sleep data were synchronized with a dedicated smartphone application and visualized as hypnograms.

Previous studies have used the Philips SmartSleep device as a portable single-channel EEG system for home-based, multi-night sleep monitoring [11,12]. The device was selected to obtain objective sleep duration in participants’ home environments with relatively low participant burden, because the study was conducted under real-world conditions among working women. Although the device provided additional sleep-related parameters, such as sleep onset latency, number of awakenings, wake time, and sleep stage information, the present analysis focused on average sleep duration as the objective sleep parameter. Average objectively measured sleep duration was calculated as the mean sleep duration across all available recording nights during the monitoring period. No prespecified minimum number of recording nights was required for inclusion; all nights for which total sleep duration was available were used to calculate the participant-level mean sleep duration. The median number of recording nights per participant was 19 nights (IQR, 15.75–21; range, 2–22 nights). Two SmartSleep device configurations were used during data collection. For 11 participants who used one device configuration, total sleep duration was not displayed in the device output and could not be reconstructed from the other available sleep parameters. Therefore, objective sleep duration was unavailable for these 11 participants, leaving 68 participants with usable objective sleep-duration data.

#### 2.2.2. Questionnaires

##### Menstrual Status

Menstrual status was assessed using a self-reported questionnaire. Participants were categorized descriptively as pre-, peri-, or post-menopausal. Menstrual status was used to describe the characteristics of the analytic sample but was not included as a primary covariate in the main regression models because of the limited sample size and the exploratory nature of the present analysis.

##### Subjective Sleep Quality

Subjective sleep quality was assessed using a self-reported questionnaire item evaluating overall sleep quality, corresponding to the subjective sleep quality component of the Pittsburgh Sleep Quality Index (PSQI) [13]. Responses were recorded on a four-point Likert scale: very poor, fairly poor, fairly good, and very good. For the primary analysis, responses were dichotomized into poor sleep quality (very poor or fairly poor) and good sleep quality (fairly good or very good), with poor sleep quality coded as 1 and good sleep quality as 0. This single-item measure was selected as the primary outcome because the present study specifically focused on participants’ overall perceived sleep quality rather than the broader multidimensional construct captured by the global PSQI score. To assess the robustness of the findings, an additional sensitivity analysis was performed using the conventional PSQI global score cutoff of >5 to define poor sleep.

##### Sleep Medication Use

Sleep medication use was assessed using the corresponding PSQI item regarding the frequency of medication use for sleep during the previous month. Responses were dichotomized into no use and any use. Sleep medication use was used to describe the sample characteristics but was not included in the main regression models because of the limited sample size.

##### Menopausal Symptoms

Menopausal symptoms were assessed using the Simplified Menopausal Index (SMI), a commonly used self-administered scale in Japan for assessing the severity of menopausal symptoms [14]. The SMI consists of 10 items categorized into three symptom domains. The vasomotor domain includes hot flashes, excessive sweating, sensitivity to cold in the lower back or extremities, and shortness of breath or palpitations. The psychological domain includes difficulty falling asleep or light sleep, irritability or nervousness, and mood swings or depressive feelings. The somatic domain includes frequent headaches, dizziness, or nausea; fatigability or easy exhaustion; and stiff shoulders, back pain, or pain in the limbs. In the primary analyses, the total SMI score and domain-specific scores were examined separately, with higher scores indicating greater symptom burden. Because the psychological domain includes a sleep-related item (“difficulty falling asleep or light sleep”), which may conceptually overlap with the subjective sleep-quality outcome, sensitivity analyses were additionally performed after excluding this item from both the total SMI score and the psychological domain score.

### 2.3. Statistical Analysis

The present analyses were based on baseline questionnaire data and objectively measured sleep duration obtained during the home monitoring period. The analytic sample was restricted to participants aged 40 years and older.

Participant characteristics were summarized using means and standard deviations for continuous variables and numbers and percentages for categorical variables. Subjective sleep quality was dichotomized into poor sleep quality and good sleep quality. Differences in menopausal symptom scores and objectively measured sleep duration between the poor sleep quality and good sleep quality groups were examined using independent-samples *t*-tests.

Logistic regression analyses were performed to examine the association between menopausal symptom burden and poor sleep quality. Poor sleep quality was coded as 1 and good sleep quality as 0. First, total SMI score was entered as the main explanatory variable, with adjustment for age. A second model additionally adjusted for objectively measured sleep duration. Domain-specific models were then constructed separately for vasomotor, psychological, and somatic symptom scores, with adjustment for age and objectively measured sleep duration. Because of the limited sample size, menopausal symptom domains were not entered simultaneously in the same model.

Odds ratios (ORs), 95% confidence intervals (CIs), and *p*-values were calculated. Statistical significance was set at *p* < 0.05. Statistical analyses were conducted using IBM SPSS Statistics version 28.0 (IBM Corp., Armonk, NY, USA).

Several sensitivity and supplementary analyses were conducted to assess the robustness of the findings and address potential confounding or measurement overlap. First, the total SMI score and psychological domain score were recalculated after excluding the sleep-related SMI item (“difficulty falling asleep or light sleep”), and the corresponding logistic regression models were repeated. Second, an additional sensitivity analysis was performed using the conventional PSQI global score cutoff (>5) to define poor sleep. Third, menopausal status (pre-menopausal, peri-menopausal, and post-menopausal) was additionally included as a categorical covariate in the regression model. Fourth, weekly working hours during the previous 7 days were additionally included as a work-related covariate. To examine whether menopausal symptoms and subjective sleep quality changed across the three questionnaire assessments, total SMI score and subjective sleep quality at baseline, Week 1, and Week 2 were compared using Friedman tests. Assumptions for parametric analyses were assessed by examining variable distributions, potential outliers, and homogeneity of variance using Levene’s test. For logistic regression analyses, multicollinearity and influential observations were assessed using variance inflation factors, standardized residuals, and Cook’s distance, and no substantial violations were identified. In addition, participants with and without available objective sleep-duration data were compared with respect to age, BMI, total SMI score, and PSQI global score using independent-samples *t*-tests.

## 3. Results

The baseline characteristics of the analytic sample are presented in Table 1. The final analytic sample included 79 Japanese working women aged 40 years and older. The mean age was 49.57 years (SD 5.20; range, 40–60 years). Regarding menstrual status, 24 participants were pre-menopausal, 19 were peri-menopausal, and 36 were post-menopausal. Poor sleep quality was reported by 43 participants, whereas 36 reported good sleep quality.

Comparisons between the good and poor sleep quality groups are presented in Table 2. The poor sleep quality group had significantly higher total SMI scores and higher vasomotor, psychological, and somatic symptom scores than the good sleep quality group. Objectively measured sleep duration was significantly shorter in the poor sleep quality group than in the good sleep quality group.

Objective sleep duration was available for 68 of the 79 participants. For the remaining 11 participants, total sleep duration could not be obtained because of a device-type-specific output limitation. Participants with and without available objective sleep-duration data did not differ significantly in age, BMI, total SMI score, or PSQI global score.

The results of the logistic regression analyses are presented in Table 3. In the age-adjusted model, higher total SMI score was significantly associated with poor sleep quality. This association remained significant after additional adjustment for objectively measured sleep duration. In domain-specific models adjusted for age and objectively measured sleep duration, vasomotor, psychological, and somatic symptom scores were each significantly associated with poor sleep quality. Objectively measured sleep duration was inversely associated with poor sleep quality across models, indicating that shorter sleep duration was associated with higher odds of poor sleep quality.

Several sensitivity analyses were performed to assess the robustness of the primary findings. The associations remained significant after excluding the sleep-related SMI item from the total SMI score and the psychological domain score, and when poor sleep was defined using the conventional PSQI global score cutoff (>5) (Table 4).

The association between total SMI score and poor sleep quality also remained significant after additional adjustment for menopausal status and weekly working hours (Table 4). Across the three questionnaire time points, no significant changes were observed in total SMI score (Friedman test, *p* = 0.072) or subjective sleep quality (*p* = 0.792). Participants with and without available objective sleep-duration data did not differ significantly in age, BMI, total SMI score, or PSQI global score.

## 4. Discussion

This exploratory cross-sectional study examined the association between menopausal symptom burden and self-reported poor sleep quality among Japanese working women aged 40 years and older, while accounting for objectively measured sleep duration. The poor sleep quality group had higher total SMI scores and higher vasomotor, psychological, and somatic symptom scores than the good sleep quality group. In logistic regression analyses, total SMI score remained associated with poor sleep quality after adjustment for age and objectively measured sleep duration. In domain-specific models, vasomotor, psychological, and somatic symptom scores were each associated with poor sleep quality.

The robustness of the primary findings was supported by several sensitivity analyses. Importantly, the associations remained significant after excluding the sleep-related SMI item from both the total SMI score and the psychological domain score, suggesting that the observed relationships were not solely attributable to conceptual overlap between the SMI sleep item and the subjective sleep-quality outcome. The association also remained significant when poor sleep was defined using the conventional PSQI global score cutoff (>5) and after additional adjustment for menopausal status and weekly working hours. In supplementary analyses comparing the three assessment points, no statistically significant differences were observed in total SMI score or subjective sleep quality; however, these findings should be interpreted cautiously because the recall periods differed between the baseline and follow-up assessments.

Importantly, the present study did not aim to determine whether menopausal symptoms cause poor sleep quality. Rather, it sought to clarify whether self-reported poor sleep quality among working women in midlife is accompanied by greater menopausal symptom burden, even when objectively measured sleep duration is considered.

These findings suggest that self-reported poor sleep quality among working women in midlife may reflect not only shorter sleep duration but also broader menopausal symptom burden. Previous studies have reported associations between menopausal symptoms and sleep problems during the menopausal transition [1,2,7]. The present study adds to this evidence by focusing on working women and by showing that menopausal symptom burden was associated with self-reported poor sleep quality even after accounting for objectively measured sleep duration. This suggests that women’s perceptions of poor sleep may not be explained by sleep duration alone and may also reflect physical and psychological symptom burden.

Among the symptom domains, psychological symptoms showed a strong association with poor sleep quality. Psychological symptoms such as irritability, anxiety, depressed mood, and emotional distress may influence both the perception of sleep quality and the sense of restorative sleep. Conversely, sleep disturbance itself may exacerbate mood and psychological symptoms, and the cross-sectional design of the present study does not allow the direction of this relationship to be determined. Importantly, the association between the psychological domain and poor sleep quality remained significant even after exclusion of the sleep-related SMI item, suggesting that this finding was not solely driven by overlap between the sleep item and the outcome measure. Previous reviews have emphasized that sleep disturbance during the menopausal transition is multifactorial and may reflect not only reproductive hormonal changes but also vasomotor symptoms, depressive symptoms, anxiety, and other physical and psychosocial factors [15,16].

Vasomotor and somatic symptoms were also associated with poor sleep quality, supporting the multidimensional nature of sleep complaints during midlife. Vasomotor symptoms, including hot flashes and sweating, may disrupt sleep continuity through nocturnal awakenings and increased wakefulness after sleep onset [17]. Recent reviews have highlighted vasomotor symptoms as an important contributor to sleep disturbance during the menopausal transition, while also noting that sleep problems may occur independently of vasomotor symptoms [15,18]. Somatic symptoms such as fatigue, headache, dizziness, musculoskeletal discomfort, and pain may further affect perceived sleep quality and daytime recovery. These findings therefore suggest that self-reported poor sleep quality in midlife women may reflect a broader constellation of psychological, vasomotor, and somatic menopausal symptoms rather than a single sleep-related problem.

The findings may have practical implications for clinical and occupational health settings. Sleep complaints are often easier for women to recognize and report than menopausal symptoms themselves. Therefore, asking about perceived sleep quality may provide a practical entry point for identifying broader menopausal symptom burden, including psychological, vasomotor, and somatic symptoms. In clinical settings, women presenting with persistent sleep complaints during midlife may benefit from further assessment of menopausal symptoms rather than evaluation of sleep duration alone. In occupational health settings, sleep-related complaints may similarly provide an opportunity for early health consultation, education, and referral to appropriate healthcare services when needed. Such an approach may facilitate earlier recognition and support before symptoms substantially affect daily functioning or work performance. However, these implications should be interpreted cautiously because of the exploratory design and limited sample size.

### Limitations of the Study

This study has several limitations. First, because of the cross-sectional nature of the present analysis, causal relationships or the directionality of associations between menopausal symptoms and poor sleep quality cannot be determined. Poor sleep quality may reflect greater menopausal symptom burden, but the reverse relationship is also possible.

Second, the primary outcome was based on a single questionnaire item assessing overall subjective sleep quality, rather than the full multidimensional PSQI construct. Therefore, the primary analysis did not capture other dimensions of sleep disturbance, such as sleep latency, sleep efficiency, nocturnal awakenings, or daytime dysfunction. However, in a sensitivity analysis using the conventional PSQI global score cutoff (>5) to define poor sleep, the association between menopausal symptom burden and poor sleep remained significant.

Third, the SMI includes a sleep-related item (“difficulty falling asleep or light sleep”) within the psychological domain, raising the possibility of conceptual overlap with the subjective sleep-quality outcome. To address this concern, sensitivity analyses were performed after excluding the sleep-related item from both the total SMI score and the psychological domain score, and the associations remained significant. Nevertheless, some conceptual overlap between menopausal symptoms and perceived sleep quality may still remain.

Fourth, the sample size was relatively small, and the analysis was restricted to Japanese working women aged 40 years and older. Objective sleep duration was available for 68 of the 79 participants. For the remaining 11 participants, total sleep duration could not be obtained because of a device-type-specific limitation in the SmartSleep output. Participants with and without available objective sleep-duration data did not differ significantly in age, BMI, total SMI score, or PSQI global score. In addition, no prespecified minimum number of recording nights was required for inclusion, which may have introduced variability in the reliability of participant-level mean sleep duration.

Fifth, although the wearable EEG device provided additional sleep-related parameters, the present analysis focused only on average objectively measured sleep duration. Other objective sleep parameters, such as sleep onset latency, nocturnal awakenings, and sleep-stage information, were not included. In addition, dedicated validation evidence for quantitative total sleep duration measurement using this specific SmartSleep configuration remains limited [19]; therefore, the objective sleep findings should be interpreted with caution.

Sixth, participants were recruited through professional and community networks, which may have introduced selection bias. Data were collected in five cohorts over approximately one year, and seasonal variation in sleep may have influenced the results. Although weekly working hours were examined in a sensitivity analysis, detailed information on shift-work status, perceived job stress, chronotype, and detailed medication use was not fully assessed or incorporated into the present analysis; therefore, residual confounding may remain.

Despite these limitations, this study simultaneously considered self-reported sleep quality, objectively measured sleep duration, and menopausal symptom burden, and the main findings remained consistent across several sensitivity analyses.

## 5. Conclusions

Self-reported poor sleep quality among Japanese working women aged 40 years and older was associated with higher menopausal symptom burden. This association remained significant after adjustment for age and objectively measured sleep duration. Vasomotor, psychological, and somatic symptom domains were each associated with poor sleep quality. These findings suggest that sleep complaints among working women in midlife may reflect not only shorter sleep duration but also broader menopausal symptom burden. Attention to self-reported sleep quality may provide a practical opportunity to recognize menopausal symptoms and support women’s health in clinical and occupational health settings.

## Figures and Tables

**Table 1:** Baseline characteristics of participants aged 40 years and older (*N* = 79).

Characteristics		Value
**Age, years**		49.57 (5.2)
**Age group**	40s	36 (45.6)
	50s	42 (53.2)
	60s	1 (1.3)
**BMI** **, kg/m** ^2^		22.56 (4.2)
**Menstrual status**	Pre-menopausal	24 (30.4)
	Peri-menopausal	19 (24.1)
	Post-menopausal	36 (45.6)
**SMI (Simplified Menopausal Index)**	Total score	32.57 (22.6)
**SMI (domain scores)**	Vasomotor symptoms score	12.14 (10.4)
	Psychological symptoms score	13.56 (10.0)
	Somatic symptoms score	6.87 (4.7)
**Sleep quality**	Very poor	2 (2.5)
	Fairly poor	41 (51.9)
	Fairly good	36 (45.6)
	Very good	0 (0.0)
**Sleep medication use**	No	70 (88.6)
	Yes	9 (11.4)
**Objective sleep duration (minutes)**		271.8 (73.0)

Values are presented as mean (SD) for continuous variables and *n* (%) for categorical variables. Objective sleep duration was available for 68 participants. BMI, body mass index.

**Table 2:** Comparison of menopausal symptom scores and objective sleep duration between good and poor sleep quality groups.

Variable	Good Sleep Quality	Poor Sleep Quality	*p*-Value
**Total SMI score**	21.72 (19.14)	41.65 (21.34)	<0.001
**Vasomotor symptoms score**	9.22 (9.18)	14.58 (10.81)	0.021
**Psychological symptoms score**	7.75 (7.68)	18.42 (9.09)	<0.001
**Somatic symptoms score**	4.75 (4.15)	8.65 (4.47)	<0.001
**Objective sleep duration, minutes**	294.52 (67.43)	251.60 (72.71)	0.014

Values are presented as mean (SD). *p* values were calculated using independent-samples *t*-tests. Objective sleep duration was available for 32 participants in the good sleep quality group and 36 participants in the poor sleep quality group. SMI, Simplified Menopausal Index.

**Table 3:** Logistic regression analyses of factors associated with poor sleep quality.

Model	Variable	OR	95% CI	*p*-Value
**Model 1**	Age	0.937	0.846–1.038	0.211
	Total SMI score	1.051	1.024–1.080	<0.001
**Model 2**	Age	0.897	0.795–1.012	0.079
	Objective sleep duration	0.989	0.981–0.998	0.012
	Total SMI score	1.053	1.023–1.084	<0.001
**Model 3**	Age	0.921	0.827–1.025	0.133
	Objective sleep duration	0.989	0.982–0.997	0.009
	Vasomotor symptoms score	1.072	1.012–1.135	0.017
**Model 4**	Age	0.897	0.789–1.019	0.095
	Objective sleep duration	0.989	0.980–0.998	0.015
	Psychological symptoms score	1.154	1.073–1.241	<0.001
**Model 5**	Age	0.880	0.779–0.995	0.041
	Objective sleep duration	0.991	0.983–0.999	0.035
	Somatic symptoms score	1.270	1.105–1.460	<0.001

Models 2–5 were based on participants with available objective sleep-duration data (*n* = 68). OR, odds ratio; CI, confidence interval; SMI, Simplified Menopausal Index. For clinical interpretability, a 10-point increase in total SMI score corresponded to approximately 1.64-fold higher odds of poor sleep quality (95% CI, 1.27–2.16). Poor sleep quality was coded as 1 and good sleep quality as 0. Model 1 was adjusted for age. Models 2–5 were adjusted for age and objectively measured sleep duration. Total SMI score and each symptom domain score were entered into separate models.

**Table 4:** Sensitivity analyses of the association between menopausal symptom burden and poor sleep quality.

Sensitivity Analysis	Main Predictor	OR	95% CI	*p*-Value
**SMI total excluding sleep-related item**	SMI total excluding sleep item	1.053	1.019–1.087	0.002
**Psychological domain excluding sleep-related item**	Psychological score excluding sleep item	1.162	1.057–1.276	0.002
**PSQI global score > 5 as outcome**	SMI total excluding sleep item	1.040	1.004–1.077	0.030
**Adjusted for menopausal status**	Total SMI score	1.049	1.019–1.080	0.001
**Adjustment for weekly working hours**	Total SMI score	1.053	1.023–1.085	<0.001

OR, odds ratio; CI, confidence interval; SMI, Simplified Menopausal Index; PSQI, Pittsburgh Sleep Quality Index. All models were adjusted for age and objectively measured sleep duration unless otherwise specified.

## Data Availability

The data presented in this study are available from the corresponding author on reasonable request. The data are not publicly available due to ethical restrictions.
